# Supplementary material for: Science in motion: A qualitative analysis of journalists’ use and perception of preprints
Source: PLoS One. 2022 Nov 21;17(11):e0277769. doi: 10.1371/journal.pone.0277769 (PMC9678308; doi:10.1371/journal.pone.0277769)
Supplement: Attachment — Submitted filename: Response to Reviewers.docx [file pone.0277769.s001.docx]

Dear reviewers,

We thank you for your feedback and suggestions regarding our manuscript entitled, “Science in motion: A qualitative analysis of journalists’ use and perception of preprints” (Manuscript PONE-D-22-04088) by Fleerackers, Moorhead, Maggio, Fagan and Alperin. We are pleased to resubmit our manuscript with revisions based on your feedback.

In the table below, we provide a description of how we addressed each of your comments. These revisions are also noted within the document using track changes. As requested, we are also submitting this marked-up copy of our manuscript, as well a revised “clean” version.

| **Reviewer comment** | **Author response** | **Edit location** |
| --- | --- | --- |
| 1. More recommendations should be suggested. (Reviewer 1) | We have added additional recommendations for practice throughout the discussion section. | Lines 471-474; 497-501 |
| 2. Authors need to include in their analysis not only the agreement between the opinions of the 19 journalists interviewed but also the discrepancies that exist between them. Reading the document, both in Table 3 and in the description on page 17, it appears that they all agree or have very similar points on the three research questions, which seems unlikely given their different academic and professional backgrounds and the variety of topics covered in the interviews. | We have reviewed Table 3 and the in-text descriptions of the results and revisited our initial coding to identify any dissenting opinions that were not reflected in the manuscript. We have integrated these dissenting perspectives within the descriptive results, but only in cases where dissenting perspectives were supported by the data. However, we have chosen not to add additional dissenting examples to Table 3, as this table is meant to provide a summary of the dominant perspectives of our participants, which we complement with the more detailed and nuanced discussion within the text itself.  Additionally, while there are multiple approaches to thematic analysis, the approach we applied in this paper (i.e., Braun & Clarke, 2012) defines themes as patterned responses or meanings that capture important aspects of the data related to the research questions. That is, the themes in this paper are not meant to represent every aspect of every individual journalist’s experiences but rather to present an overview—or “story” (Braun & Clarke, 2012)—of the key issues and perspectives that are shared among many of the journalists. | Lines 276-279; 337-338; 361-364 |
| 3. Authors need to be clearer about why is it important that we know what they are proposing, there does not seem to be a clear discussion with other authors about their findings, from the literature review conducted, do their results prove or contradict what has been said by any other author(s)? | We have more clearly articulated the importance of this research and the gaps which it seeks to fill in the Introduction. We have also revised our Conclusion to more directly engage with the PNS literature from our literature review, highlighting how our findings contribute to this body of work. | Lines  51-58;  559-582 |
| 4. Authors could make suggestions about the key challenges and issues that post-normal science communication would face in the post-COVID-19 pandemic. | Because this research was conducted in the COVID-19 context, we do not feel confident predicting key challenges and issues that PNSC might raise post-pandemic. However, in our revised conclusion, we have provided some discussion about the degree to which our findings might play out similarly in other post-normal contexts, including a call for more research examining potential challenges of PNSC in the post-pandemic world. | Lines 569-582 |

We greatly appreciate your further review of our manuscript and hope that the revisions as described above and in the revised paper meet your expectations. Please do not hesitate to contact me with any questions or additional requests.

Sincerely,

Alice Fleerackers

PhD Candidate, Interdisciplinary Studies

Simon Fraser University
